# *Abiotrophia Defectiva* as a Rare Cause of Mitral Valve Infective Endocarditis With Mesenteric Arterial Branch Pseudoaneurysm, Splenic Infarction, and Renal Infarction: A Case Report

**DOI:** 10.3389/fmed.2022.780828

**Published:** 2022-03-11

**Authors:** Jiayu Li, Li Zhou, Xuhe Gong, Yuan Wang, Daokuo Yao, Hongwei Li

**Affiliations:** ^1^Department of Cardiology, Beijing Friendship Hospital, Capital Medical University, Beijing, China; ^2^Department of Internal Medical, Medical Health Center, Beijing Friendship Hospital, Capital Medical University, Beijing, China; ^3^Beijing Key Laboratory of Metabolic Disorder Related Cardiovascular Disease, Beijing, China

**Keywords:** *Abiotrophia defectiva*, infective endocarditis, mitral valve replacement, pseudoaneurysm, case report

## Abstract

**Introduction:**

*Abiotrophia defectiva* (*A. defectiva*) is a rare species leading to infective endocarditis (IE) with a poor prognosis. We describe a previously healthy patient with mitral valve infective endocarditis caused by *A. defectiva*.

**Case report:**

A young man was admitted with intermittent fever. Echocardiography confirmed vegetation on the mitral valve with evidence of valve perforation and severe mitral regurgitation. Three sets of blood cultures became positive for *A. defectiva*. As he presented with manifestations of mesenteric arterial branch pseudoaneurysm, splenic and renal infarction, mitral valve replacement, and embolization of superior mesenteric aneurysm were operated during 8 weeks' targeted antibiotic therapy.

**Conclusion:**

This case study emphasizes the importance of considering *A. defectiva* as a rare but important cause of IE and of performing blood culture to make its accurate diagnosis and timely anti-infective treatment. Early surgical management and active prevention of complications have been associated with a favorable prognosis.

## Introduction

*Abiotrophia defectiva* (*A. defectiva*), once considered as a nutritionally variant *Streptococci* (NVS) and later moved to a new genus *Abiotrophia*, is a part of the normal flora of the oral cavity, gastrointestinal tract, and genitourinary system. Among a variety of severe invasive diseases caused by *A. defectiva*, IE is one of the most frequently reported infections. But, it is still rare for all the endocarditis, with a high rate of failure and complications in some reported cases. Thus far, a suitable treatment strategy remains to be explored.

## Case Description

A 24-year-old male patient, without preexisting diseases, was admitted for 20 days of intermittent fever (up to 38.6°C), accompanied by headache and fatigue, but no chills, cough, sputum, abdominal pain, diarrhea, chest pain, or tightness. He denied a prior history of congenital heart disease, alcoholic drinking, cigarette smoking, and injection drug use. He had not recently undergone any sore throat, dental procedures, skin rash, or surgery. He had been prescribed a non-steroidal anti-inflammatory drug by his general practitioner but this provided no relief.

Before admission, the patient had been admitted to the respiratory department in the local hospital because of fever and the laboratory studies showed normal leukocytes but elevated C-reactive protein of 38.67 mg/l (normal range: 0–8 mg/l). A transthoracic echocardiogram (TTE) revealed vegetation on the anterior aspect of the mitral leaflet, measuring 1.3 × 0.7 cm. After following the treatment with intravenous amoxicillin (dosage unknown) only once during the emergency care for 1 day, the patient was then referred to our hospital and hospitalized for further examination and treatment.

**Figure 1:**
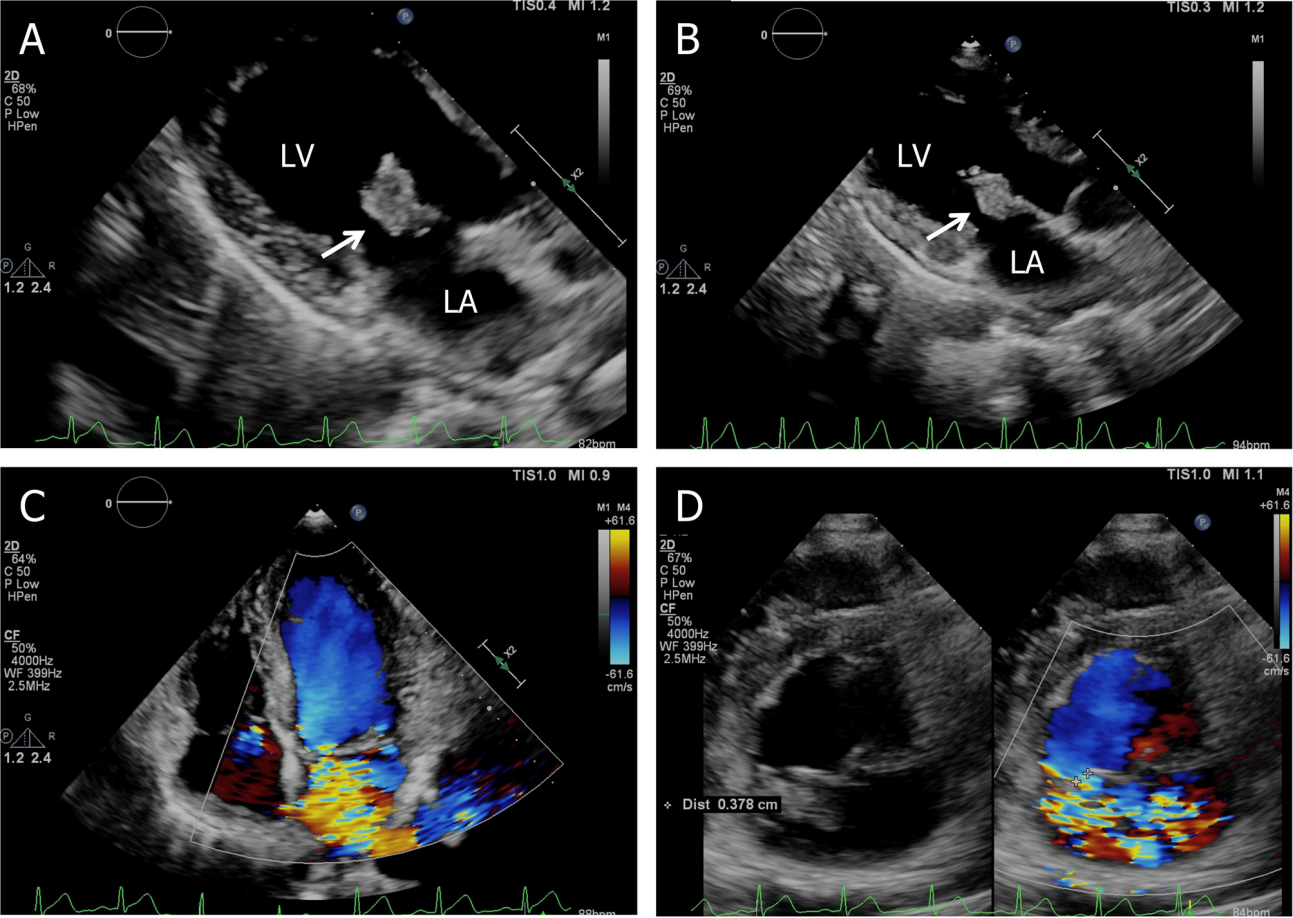
Transthoracic echocardiogram. **(A)** Apical three-chamber view shows a 2.24 cm^2^ vegetation in the anterior leaflet of the mitral valve, **(B)** the size of the vegetation decreased to 1.91 cm^2^ on the 7th hospital day, **(C)** apical four-chamber view shows severe mitral regurgitation, and **(D)** short-axis parasternal view shows severe mitral regurgitation contrasted with color Doppler. LA, left atrium; LV, left ventricle.

On admission, with a presumptive diagnosis of IE, physical examination showed a grade 4/6 systolic murmur over the mitral area upon auscultation and obvious splenomegaly *via* palpation. No Janeway's lesions, Osler's nodes were observed. Laboratory studies revealed normocytic anemia (hemoglobin 10.8 g/dl, normal range: 13.0–17.5 g/dl), normal leukocytes but elevated C-reactive protein of 30.61 mg/l, and erythrocyte sedimentation rate of 38 mm in the first hour. TTE confirmed isolated vegetation of 2.24 cm^2^ on the anterior mitral valve leaflet, with valve perforation and severe mitral regurgitation (Figure 1).

**Figure 2:**
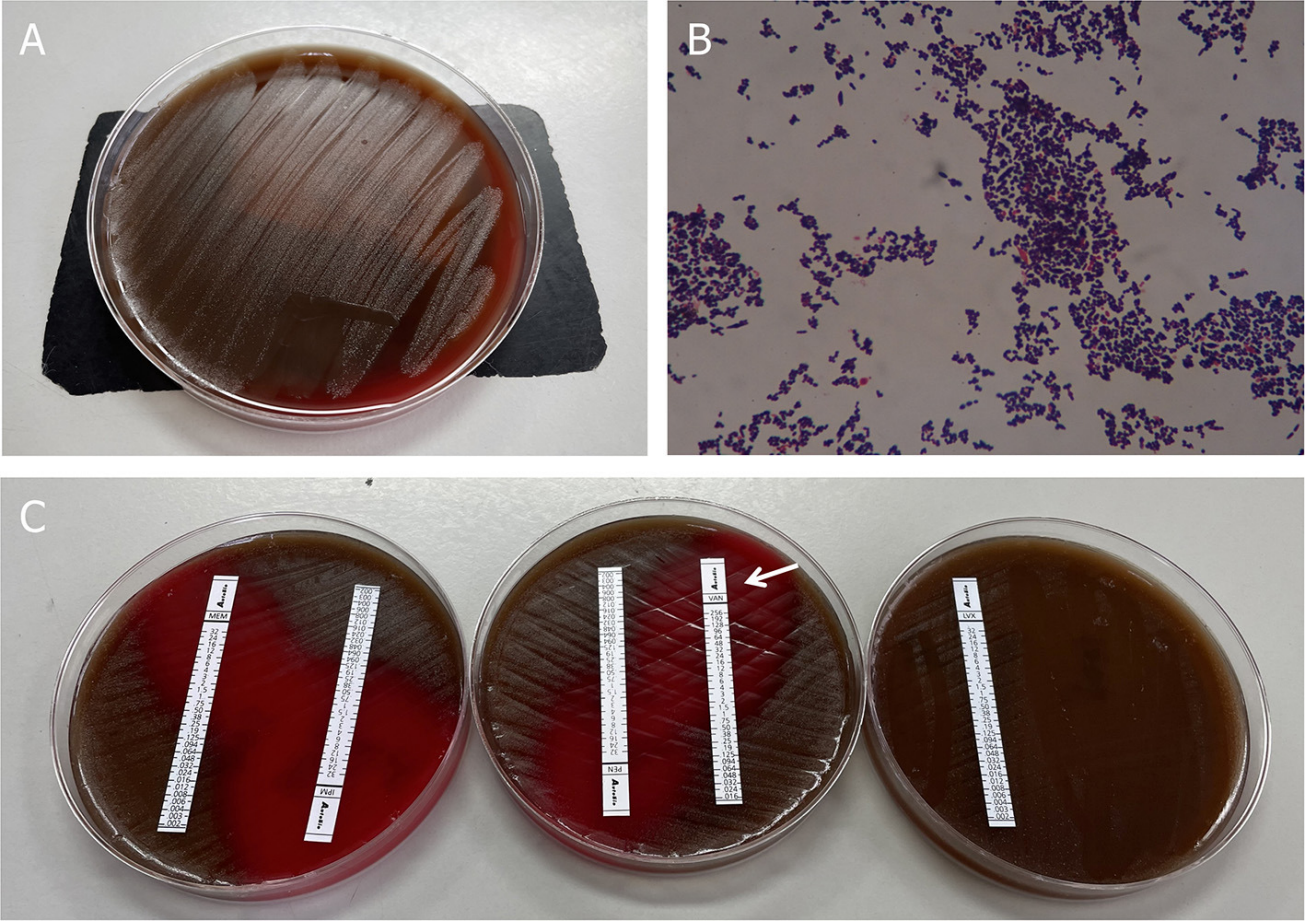
Blood culture results. **(A)** Growth of colonies of *Abiotrophia defectiva* in the blood agar. **(B)** Gram stain reveals Gram-positive pleomorphic coccobacilli, arranged in chains (original magnification, 1,000X). **(C)** Antimicrobial susceptibility testing demonstrated susceptibility to vancomycin, imipenem, and meropenem, but was resistant to penicillin and levofloxacin.

Three sets of blood cultures were taken before the patient was commenced on empirical treatment with intravenous vancomycin (1 g every 12 h). After 31 h, his blood culture became positive for Gram-positive cocci, which was identified as *Abiotrophia defectiva*. Antimicrobial susceptibility testing revealed that it is susceptible to vancomycin, imipenem, and meropenem, but resistant to penicillin and levofloxacin. All outcomes of the three blood cultures presented the same bacteria—*A. defectiva* (Figure 2).

Three sets of blood cultures were taken before the patient was commenced on empirical treatment with intravenous vancomycin (1 g every 12 h). All three sets of blood cultures consist of one bacterial aerobic flask, one bacterial anaerobic flask, and one fungal culture flask. After 31 h, his blood culture became positive for Gram-positive cocci, which was identified as *Abiotrophia defectiva* (BACTEC FX400 Automatic Bacterial Cultivator). Within the next 1–2 days, all the flasks are positive for *A. defectiva* and mass spectrometry (VITEK MS) was used to identify its authenticity. Antimicrobial susceptibility testing (Epsilometer test, E-test) revealed it susceptible to vancomycin [minimum inhibitory concentration (MIC) 0.32 ug/ml; breakpoint 100%], imipenem, and meropenem, but resistant to penicillin and levofloxacin. The MICs are assessed according to criteria listed in CLSI M45 (Figure 2, Supplementary Data 1).

Until now, the patient met the modified Duke diagnostic criteria of IE, including two main criteria (positive blood cultures and evidence of endocardial involvement) (1).

**Figure 3:**
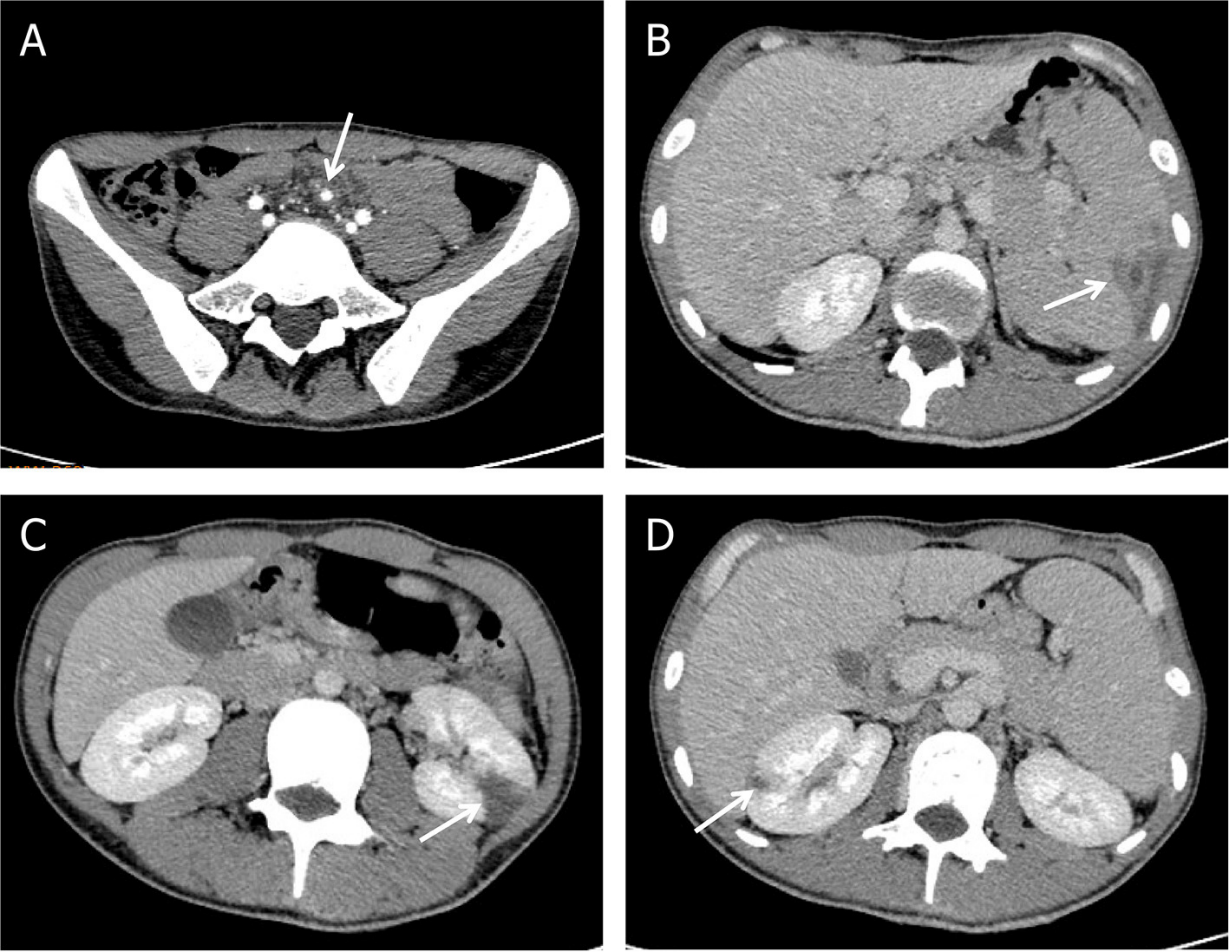
Complications of *Abiotrophia defectiva* infective endocarditis. **(A)** Mesenteric arterial branch aneurysms in abdomen CT. **(B)** Splenic infarction in abdomen CT. **(C,D)** Multiple renal infarction in abdomen CT.

While during the therapy, he developed progressively worsening abdominal pain with left upper quadrant tenderness to palpation. Contrast-enhanced abdomen CT demonstrated mesenteric arterial branch aneurysms and splenic and renal infarcts (Figure 3).

**Figure 4:**
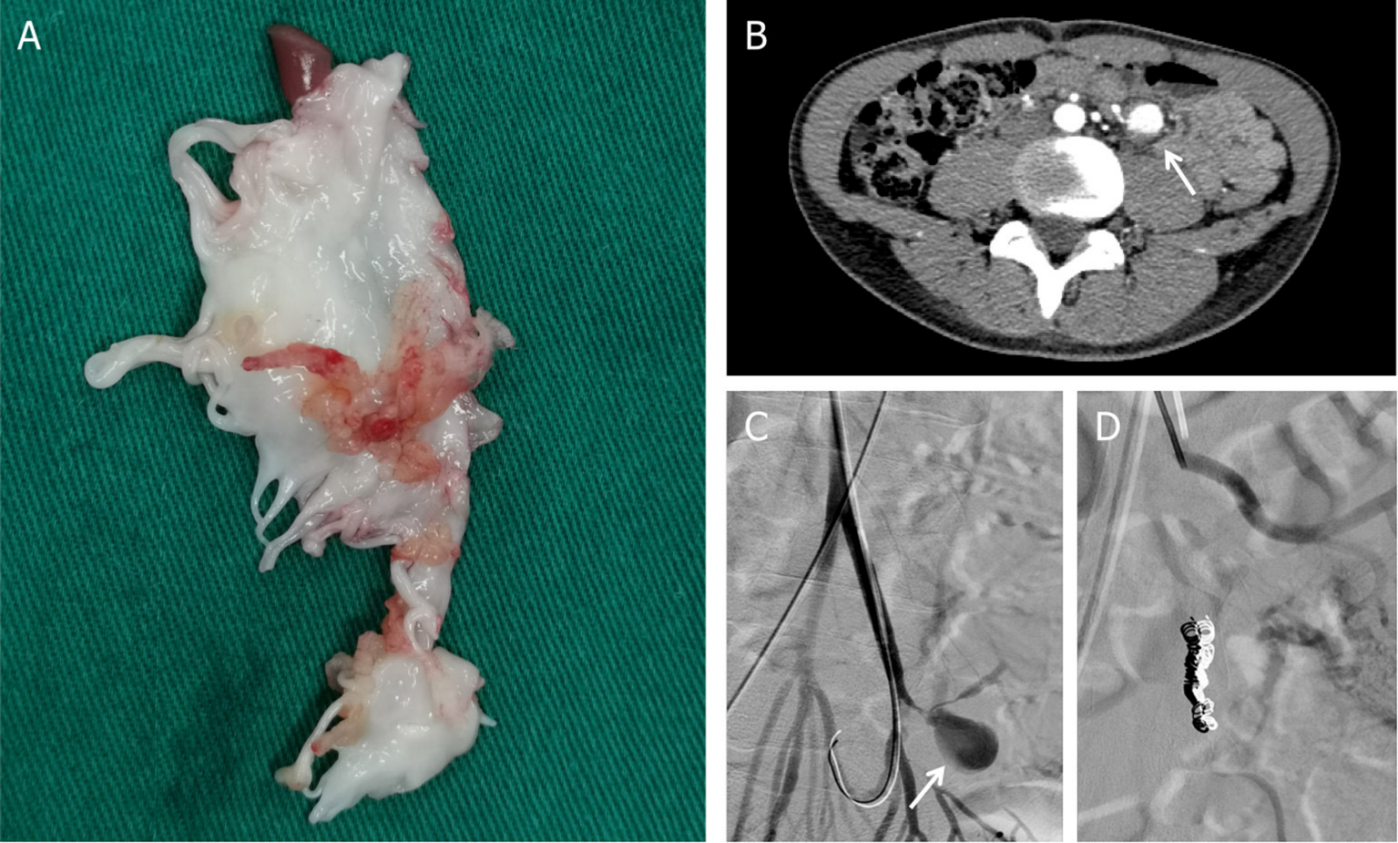
**(A)** Postoperative mitral valve specimen. **(B)** Mesenteric arterial branch aneurysms in abdomen CT. **(C)** Digital subtraction angiography (DSA) indicated pseudoaneurysms (arrow) at the mesenteric arterial branch. **(D)** Two detachable coils were used to embolize the pseudoaneurysm.

Later on hospital day 10, in case of severe complications, the patient underwent mitral valve replacement under pump (Figure 4). Intraoperatively, vegetations to the anterior leaflet of the mitral valve were seen with valve perforation. The native valve was successfully replaced with a mechanical valve (Carbomedics Prosthetic Heart Valve 27#, Sorin Group Italia Srl, Italy). Though both the cultures of vegetation and the blood after the surgery were negative, anti-infective treatment was continued with vancomycin for a planned duration of 8 weeks. The patient was started on warfarin 1.5 mg once daily for his mechanical valve.

During postoperative anti-infective treatment, repeated CT clarified no significant changes of organs' infarction but remarkable enlargement of the pseudoaneurysm with the thickened wall of the mesenteric arterial branch. To prevent the rupture of the aneurysm, two detachable coils were used to embolize the branches of the artery *via* digital subtraction angiography on hospital day 50 (Figure 4). The patient was discharged in a very good condition after 8 weeks' targeted antibiotic therapy.

**Figure 5:**
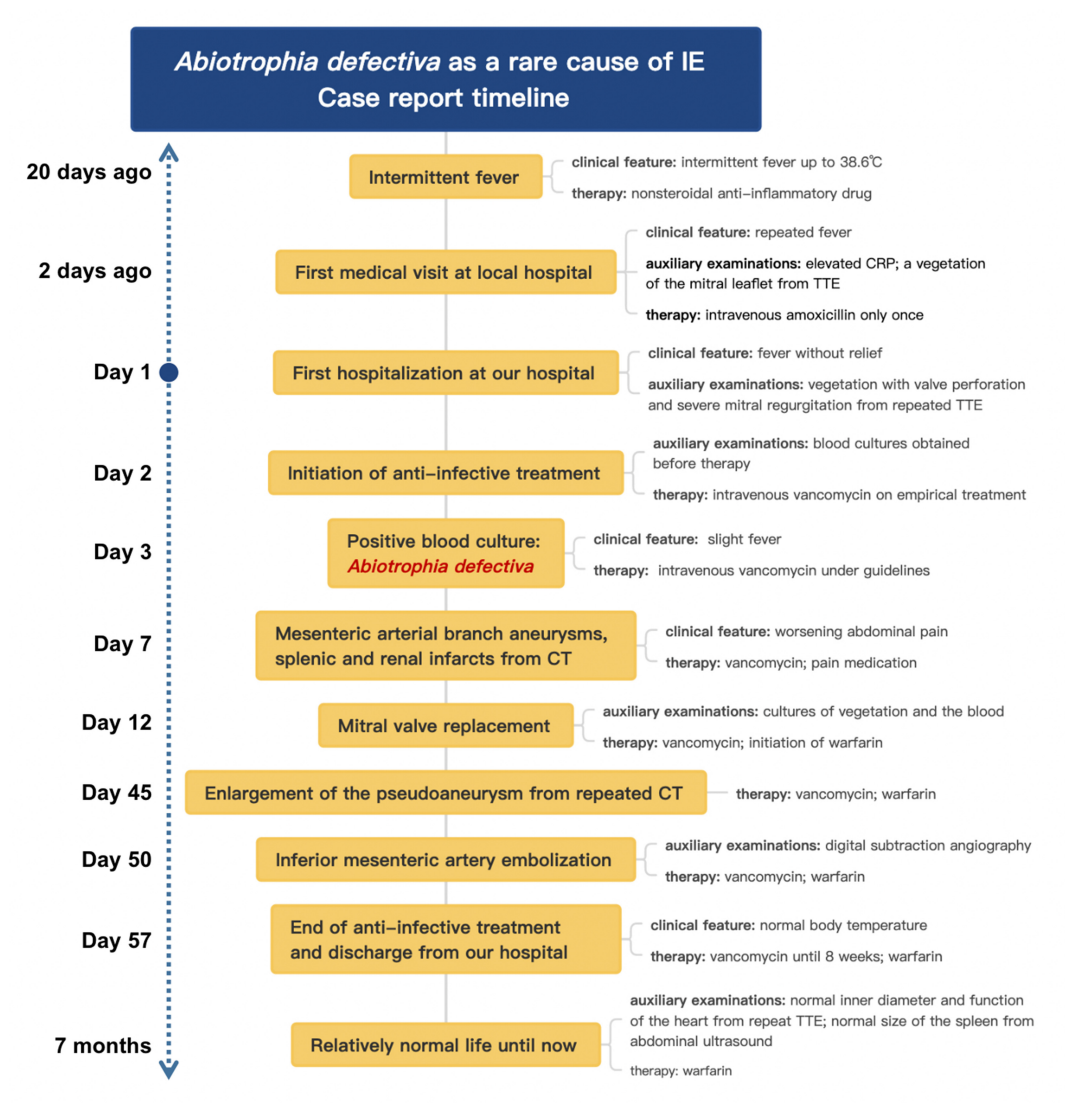
The timeline of the case.

Until 6 month-following up after discharge, he is currently symptoms free. Repeat TTE showed normal inner diameter and function of his heart. Abdominal ultrasound also showed that the spleen returned to normal size, and the infarcts gradually improved. Even with a long-term oral warfarin anticoagulation therapy, he lived a relatively healthy life. The progress and decision-making of the case above are reflected in the timeline (Figure 5).

## Discussion

As a facultative anaerobic gram-positive coccus, *A. defectiva* is first described as an NVS in 1961 from a case of infectious endocarditis (2). NVS were named the genus *Streptococcus* in 1989 following the use of 16S ribosomal RNA gene sequencing (3) and *A. defectiva* was added to the new genus *Abiotrophia* in 1995. Since the others were reclassified into a new genus, only the *A. defectiva* remained in this genus in 2000 (4). It is a component of the normal flora of the respiratory, urogenital, and gastrointestinal tracts. However, under immunocompromised conditions, *A. defectiva* was reported to cause ocular infections, otitis and sinus infections, osteoarticular and prosthetic joint infections, cerebral abscesses, iatrogenic meningitis, and pancreatic abscesses. Among these, endocarditis is the most common disease caused by *A. defectiva*. In addition, *Abiotrophia* endocarditis occupies about 4.3–6% of all streptococcal endocarditis before transferring into a new genus (5), which is characterized by a slow and indolent course. Most cases are likely misdiagnosed as culture-negative endocarditis, leading to delayed treatment. Berge et al. compared the epidemiology for IE in several streptococcus-like bacteria (SLB) among 568 episodes of bacteremia, focusing on *Abiotrophia, Aerococcus, Gemella*, and *Granulicatella*. The incidence of bacteremia with the four SLB genera was 30 episodes/1,000,000 population per year. *Abiotrophia* had the highest propensity of IE (4 of 19, 21%) and *A. defectiva* was more likely to cause IE [odds ratio (OR) 7.5, 95% CI 2.2–25, *P* < 0.05] (6).

In the etiological analysis, we also compared previously reported cases related to *A. defectiva*. Most of them have underlying heart diseases, such as rheumatic heart disease (7), congenital heart disease (8, 9), or a history of intravenous injection (10). Complications such as embolism and valve perforation almost appeared in all cases, and even multiple valves can be involved (11). Cases denying past medical history are rare, from children or non-elderly people. In these cases, serious complications such as heart failure and embolism did not occur. Therefore, simple anti-infective therapy or elective surgery after anti-infective therapy is sufficient. On the other hand, our patient, despite denying the past medical history, developed complications such as organ embolism and aneurysm in the early stage of anti-infection. In contrast, his condition was more urgent.

The choice of antibiotics in the early stage is essential. According to the current ESC guideline-recommended antibiotic therapy, penicillin G, ceftriaxone, or vancomycin should be used for 6 weeks, combined with an aminoglycoside for at least the first 2 weeks (12). Almost all susceptibility studies have also reported that *A. defectiva* is susceptible to vancomycin (13, 14). In this case study, the patient had no symptoms at first other than fever. Empirical treatment with vancomycin seems feasible through body temperature control and the decline of infectious markers. This was later confirmed by antimicrobial susceptibility testing.

Surgery is essential in the treatment of IE and is required in approximately half of the cases with severe complications (15). To prevent systemic embolism and avoid cardiac dysfunction such as heart failure and valve structural damage, patients with severe valve disease and large vegetation should consider early surgery while receiving antibiotic treatment. Both the recommendations for timing of surgery in complicated left-sided infective endocarditis from guidelines of the American College of Cardiology/American Heart Association (ACC/AHA) in 2020 (16) and the European Society of Cardiology (ESC) in 2015 (12) pointed out that early or urgent surgery should be performed if one of the following items is present: persistent bacteremia or fever lasting 5 days after onset of appropriate antibiotic therapy, recurrent emboli, and persistent vegetations despite appropriate antibiotic therapy, left-sided valve IE who exhibit mobile vegetations 10 mm in length with or without clinical evidence of the embolic event. After discussions of multiple disciplines team including experts from cardiac surgery, cardiology, vascular surgery, and infectious diseases, our patient, with a vegetation size of >10 mm, suffered from splenic and renal infarction suggesting that he might have an early mitral valve replacement. At the same time, we communicated with the patient timely about his condition, surgical risks, and costs. He agreed with the valve replacement surgery, which is closely related to his relatively enlightened thinking mode and the positive attitude toward life.

Infectious aneurysms, easier to rupture and hemorrhage with the thin walls, result from septic arterial embolism to the intraluminal space or spread of infection through the intimal vessels. There is no definite management plan for pseudoaneurysm owing to IE. While among antibiotic treatment, endovascular intervention, and surgery, it depends on the occurrence of rupture, the location in the artery bed, and the clinical status of the patient, not only the size of aneurysms (17). For the patient in this case study, embolization therapy was exerted to achieve a less invasive possibility.

In this case study, the accurate and timely identification of *A. defectiva* is of great significance for clinical diagnosis and treatment. Early surgical management and active prevention of complications reduce as many adverse events as possible. By reporting this, we wish to share our uncommon experience and hope that it may be helpful in future cases.

## Conclusion

This case study indicates that although *Abiotrophia defectiva* is a rare cause of infective endocarditis, it may lead to a higher risk of adverse events such as infarctions and aneurysms. Thus, timely detection of blood culture and early diagnosis of *A. defectiva* help to guide antibiotic therapy. Prompt imaging evaluation and surgical intervention should be scheduled to avoid late complications. A more systematic scientific study would be necessary to evaluate its characteristics in the future.

## Data Availability Statement

The original contributions presented in the study are included in the article/Supplementary Material, further inquiries can be directed to the corresponding authors.

## Ethics Statement

Written informed consent was obtained from the individual(s) for the publication of any potentially identifiable images or data included in this article.

## Author Contributions

JL, LZ, XG, DY, and HL provided clinical care for the patient. JL wrote the manuscript. YW, DY, and HL reviewed the manuscript. All the authors approved the final draft of the manuscript and have made an intellectual contribution for publication.

## Funding

This case study was supported by the National Natural Science Foundation of China (Grant No. 82070357).

## Conflict of Interest

The authors declare that the research was conducted in the absence of any commercial or financial relationships that could be construed as a potential conflict of interest.

## Publisher's Note

All claims expressed in this article are solely those of the authors and do not necessarily represent those of their affiliated organizations, or those of the publisher, the editors and the reviewers. Any product that may be evaluated in this article, or claim that may be made by its manufacturer, is not guaranteed or endorsed by the publisher.
